# Supplementary material for: Nicotinamide Prevents Apolipoprotein B-Containing Lipoprotein Oxidation, Inflammation and Atherosclerosis in Apolipoprotein E-Deficient Mice
Source: Antioxidants (Basel). 2020 Nov 21;9(11):1162. doi: 10.3390/antiox9111162 (PMC7700561; doi:10.3390/antiox9111162)
Supplement: Supplementary file 1 [file antioxidants-09-01162-s001.zip › 20201030_Supplementary_Figure_legends.doc]

**Nicotinamide prevents apolipoprotein B-containing lipoprotein oxidation, inflammation and atherosclerosis in apolipoprotein E-deficient mice** Méndez-Lara KA et al.

**Supplementary Figures**

**Supplementary Figure 1. Schemes of the experimental designs used to evaluate the effect of NAM on the development of atherosclerosis.** Two-month-old mice were challenged with a Western diet (abbreviated as HFD in the graphical image) and NAM treatments for 1 month. The mice were randomly distributed into three experimental groups. Analyses of gross, biochemical, histological and molecular parameters were performed at the end of the study. An untreated group of mice that became obese after the consumption of the HFD was used to compare the effect of the NAM manipulation.

**Supplementary Figure 2. Analysis of atherosclerosis in proximal aortas of ApoE-deficient mice treated with different doses of NAM.**

(a) Median sizes of atherosclerotic lesions in 4 consecutive sections of the proximal aorta from different groups of male ApoE-deficient mice. (b) Representative images of proximal aortic lesions in 12-week-old female mice. (c) Median atherosclerotic lesion area in the proximal aorta of female ApoE-deficient mice. (d) Median sizes of atherosclerotic lesions in 4 consecutive sections of the proximal aorta from different groups of female ApoE-deficient mice. In panels (a), (c), and (d), data were expressed as medians (interquartile ranges) (n=8 mice per group). Statistically significant differences among groups for each variable were determined using a nonparametric a Kruskal Wallis followed by Dunn’s posttest. Differences were considered significant when P<0.05. Specifically, *P<0.05 vs. Untreated group Abbreviations used: NAM LD, low-dose, NAM-treated mice; NAM HD, high-dose, NAM-treated mice; NAM, nicotinamide.

**Supplementary Figure 3. Correlations between the levels of OxLDL and cytokines in the thoracoabdominal aorta with atherosclerotic lesions in the proximal aorta of ApoE-deficient mice.** (a) Correlation between serum oxLDL and proximal aortic atherosclerotic lesion area (n=8 mice per group). (b) Correlation between aortic IL-10 abundance and proximal aortic atherosclerotic lesion area (n=5-6 mice per group). (c) Correlation between aortic TNFα abundance and proximal aortic atherosclerotic lesion area (n=5 mice per group). A nonparametric Spearman test was used to analyze the relationship between variables. All mice were considered for analysis. Differences were considered significant when P<0.05.

**Supplementary Figure 4. Effect of NAM on the mRNA expression of molecular targets involved in cholesterol transport in the thoracoabdominal aorta of ApoE-deficient mice and in cultured macrophages.** (a) Aortic mRNA levels of different cholesterol transporters and intermediates in the LXR signaling pathway. Two-month-old mice were challenged with a Western diet and NAM treatments for 1 month. Data were expressed as medians (interquartile ranges) (n=5-6 group). (b) Correlation between proximal aortic atherosclerotic lesion area and aortic *Abca1* mRNA. (c) Correlation between proximal aortic atherosclerotic lesion area and aortic *Abcg1* mRNA. (d) Relative levels of the *Abca1* and *Abcg1* mRNAs in LPS-activated J774A.1 macrophages exposed to different doses of NAM for 24 h. (e) HDL-mediated cholesterol efﬂux from NAM-pretreated J774A.1 macrophages. Human HDL (50 μg cholesterol/mL) isolated from plasma were added to the culture medium of J774A.1 cells loaded with [^3^H]-cholesterol and preincubated at the indicated concentrations of NAM. The percentage of cholesterol efﬂux was determined. In panels (a), (d), and (e), statistically significant differences among groups for each variable were determined using a nonparametric a Kruskal Wallis followed by Dunn’s posttest. In panels (d) and (e), data were representative of 3-4 independent experiments. Differences were considered significant when P<0.05. Specifically, *P<0.05 vs. untreated group or † P<0.05 vs. NAM HD-treated mice. In panels (c) and (d), the relationship between parameters was tested using a nonparametric Spearman’s correlation test. In panel (c), mice of all groups were considered for analysis. Abbreviations used: NAM LD, low-dose, NAM-treated mice; NAM HD, high-dose, NAM-treated mice; NAM, nicotinamide.

**Supplementary Figure 5. Negative controls for immunohistochemical staining and area fraction calculation with ImageJ software.** (a) Representative images of negative controls for the IL-10 and TNFα staining shown in Figure 3, panel (e) in thoracoabdominal aortas from NAM HD-treated mice. Negative controls for the data shown in Figure 3 were performed for IL-10 and TNFα in different control blocks including aortic tissue incubated with the respective secondary antibodies used for immunostaining (of which a cutout is shown).
