# Supplementary material for: Nicotinamide Prevents Apolipoprotein B-Containing Lipoprotein Oxidation, Inflammation and Atherosclerosis in Apolipoprotein E-Deficient Mice
Source: Antioxidants (Basel). 2020 Nov 21;9(11):1162. doi: 10.3390/antiox9111162 (PMC7700561; doi:10.3390/antiox9111162)
Supplement: Supplementary file 1 [file antioxidants-09-01162-s001.zip › 20201030_Supplementary_Tables.doc]

**Nicotinamide prevents apolipoprotein B-containing lipoprotein oxidation, inflammation and atherosclerosis in apolipoprotein E-deficient mice**

Méndez-Lara KA et al.

**Supplementary Tables**

Supplementary Table 1. Transitions used in QqQ shown by different metabolites.

| Metabolite | RT (min) | 1st Transition (CE (V)) | 2nd Transition (CE (V)) |
| --- | --- | --- | --- |
| N-Me-Nicotinamide | 0.76 | 137 → 80 (24) | 137 → 108 (16) |
| d_4_-Nicotinamide (IS) | 0.91 | 127 → 84 (24) | 127 → 81 (28) |
| Nicotinamide | 0.93 | 123 → 80 (20) | 123 → 53 (32) |

Supplementary Table 2. List of specific Taqman probes used for gene expression analysis

| Gene | Protein | Reference |
| --- | --- | --- |
| *Abca1* | ATP-binding cassette, sub-family A, member 1 | Mm00442646_m1 |
| *Abcg1* | ATP-binding cassette, sub-family G, member 1 | Mm00437390_m1 |
| *Il1b* | Interleukin 1 beta | Mm00434228_m1 |
| *Il10* | Interleukin 10 | Mm01288386_m1 |
| *Il6* | Interleukin 6 | Mm00446190_m1 |
| *Tnfa* | Tumor necrosis factor | Mm99999068_m1 |

Supplementary Table 3. List of primers sequences used for gene expression analysis by SYBR green

| Gene | 5' Primer | 3' Primer |
| --- | --- | --- |
| *Abca1* | GCGAGGGCTCATCGACAT | GAAGCGGTTCTCCCCAAAC |
| *Abcg1* | TCACCCAGTTCTGCATCCTCTT | GCAGATGTGTCAGGACCGAGT |
| *Adgre1* | CTTTGGCTATGGGCTTCCAGTC | GCAAGGAGGACAGAGTTTATCGTG |
| *Il10* | AGCCTTATCGGAAATGATCCAGT | GGCCTTGTAGACACCTTGGT |
| *Nr1h3* | CCTTCCTCAAGGACTTCAGTTACAA | CATGGCTCTGGAGAACTCAAAGAT |
| *Nr1h2* | CATTGCGACTCCAGGACAAGA | CCCAGATCTCGGACAGCAAG |
| *Tnfa* | CCAGACCCTCACACTCAGATC | CACTTGGTGGTTTGCTACGAC |
| *L14* | TCCCAGGCTGTTAACGCGGT | GCGCTGGCTGAATGCTCTG |

Supplementary Table 4. Characteristics of primers used gene expression analysis by SYBR green

|  | Sequence accession number |  | Location of each primer by exon | In silico specificity screen | Amplicon length (bp) | Targeted splice variants | Software (source, version) |
| --- | --- | --- | --- | --- | --- | --- | --- |
| *Abca1* | NM_013454.3 | Fw | Exon 40 | Primer-Blast | 73 | Transcript variants X1-X3 | Designed with Primer3web version 4.0.0 |
|  |  | Rv | Exon 41 |  |  |  |  |
| *Abcg1* | NM_009593.2 | Fw | Exon 10 | Primer-Blast | 72 | NM_009593.2 | Designed with Primer3web version 4.0.0 |
|  |  | Rv | Exon 11 |  |  |  |  |
| *Adgre1* | NM_001355722 | Fw | Exon 17 | Primer-Blast | 165 | Transcript variants 1-3, X1 | Designed with Primer3web version 4.0.0 |
|  |  | Rv | Exon 19 |  |  |  |  |
| *Il10* | NM_010548.2 | Fw | Exon 3 | Primer-Blast | 229 | NM_010548.2 | PrimerBank database; ID: 291575143c3 |
|  |  | Rv | Exon 5 |  |  |  |  |
| *Nr1h2* | NM_009473.3 | Fw | Exon 10 | Primer-Blast | 51 | Transcript variants 1-4, X1-X4 | Designed with Primer3web version 4.0.0 |
|  |  | Rv | Exon 10 |  |  |  |  |
| *Nr1h3* | NM_013839.4 | Fw | Exon 7 | Primer-Blast | 98 | Transcript variants 1-3, X1-X3 | Designed with primer3web version 4.0.0 |
|  |  | Rv | Exon 8 |  |  |  |  |
| *Tnfa* | NM_013693.3 | Fw | Exon 2 | Primer-Blast | 79 | Transcript variant 1 | Designed with Primer3web version 4.0.0 |
|  |  | Rv | Exon 4 |  |  |  |  |
| *L14* | NM_026732.3 | Fw | Exon 1 | Primer-Blast | 101 | NM_026732.3 | Designed with Primer3web version 4.0.0 |
|  |  | Rv | Exon 2 |  |  |  |  |
