# Supplementary material for: Nicotinamide Prevents Apolipoprotein B-Containing Lipoprotein Oxidation, Inflammation and Atherosclerosis in Apolipoprotein E-Deficient Mice
Source: Antioxidants (Basel). 2020 Nov 21;9(11):1162. doi: 10.3390/antiox9111162 (PMC7700561; doi:10.3390/antiox9111162)
Supplement: Supplementary file 1 [file antioxidants-09-01162-s001.zip › antioxidants-1002260-R1-b_suppl._methods.doc]

**Nicotinamide prevents apolipoprotein B-containing lipoprotein oxidation, inflammation and atherosclerosis in apolipoprotein E-deficient mice**

Méndez-Lara KA et al.

**Supplementary Methods**

*Mice and treatments*

All animal procedures were reviewed and approved by the Institutional Animal Care Committee and Use Committee of the Institut de Recerca de l’Hospital de la Santa Creu i Sant Pau (Procedure Nº 10434). The methods were conducted in accordance with the approved guidelines. The effect of nicotinamide (NAM) (cat#N0636, Merck KGaA, Darmstadt, Germany) supplementation on male ApoE-deficient mice was examined. ApoE-deficient mice were on a C57BL/6J background and acquired from Jackson Laboratories (Bar Harbor, ME, USA; stock number 02052. <https://www.jax.org/strain/002052>). Animals had free access to food and water and were used after a minimum of 7 days acclimatization to the housing conditions. Animals were kept in a temperature-controlled environment (20ºC) and humidity (66%) with a 12-h light/dark cycle. Two doses of NAM (high dose: 1%; low dose: 0.25%) (cat#N0636, Merck KGaA, Darmstadt, Germany) were administered to mice in the drinking water, starting at the same time as the high-fat diet was initiated. The design of the intervention is shown in **Figure S1**. Two-month-old, male mice were randomly distributed into three groups (n = 8 mice each, n = 24) depending on whether they received tap non-supplemented water (untreated) or NAM supplementation (i.e., 0.25% and 1%). All mice were, at the same time challenged with a high-cholesterol, high-fat diet (western diet) (TD.88137, ENVIGO, containing 21% of fat by weight, and 27% saturated fat/total fat, and 0.25% cholesterol) for 1 month.

Food and water consumption was weekly monitored during the period of treatment. The calculated intake of NAM (g/mouse/day) in treated mice is shown in **Table 1**. Similar experimental design was also pursued in female mice to assess sex-related differences at the maximal NAM dose (**Figure S2**). At the end of the studies, the mice were individually placed in regular cages and acclimatized for 24 h and food and water intake monitored for the next two days prior euthanasia. Food-deprived (4 h) mice were euthanized by ex-sanguination by cardiac puncture after being anesthetized with isoflurane vapors (Forane®, Abbott).

*Plasma lipids and lipoprotein analysis*

The biochemical parameters were analyzed using commercial kits and adapted to a COBAS 6000/c501 autoanalyzer (Roche Diagnostics, Basel, Switzerland), as described in a previous study [1]. Hepatic triglycerides were extracted after solvent extraction [2] and determined as above.

*Cytokine determination in plasma using a multiplex approach*

Plasma concentrations of IL-10, IL-6, IL-4, and TNFα were analyzed using a comprehensive customized, magnetic bead-based Milliplex Map Mouse Cytokine/Chemokine Magnetic Bead assay kit (cat#MCYTOMAG-70K; Merck-Millipore) in a Luminex Corporation® MAGPIX® instrument with XPONENT® using xMAP® technology (Millipore Corporation, Billerica, MA).

*NAM and me-NAM determination in plasma*

NAM and me-NAM levels were analyzed using high-performance liquid chromatography (HPLC) with mass spectrometry (MS). Plasma was extracted with cold acetonitrile:methanol:water (5:4:1, v:v:v) and analyzed by ultra-high-performance liquid chromatography system coupled to a 6490 triple quadrupole mass spectrometer (Agilent Technologies) with electrospray ion source (LC-ESI-QqQ) working in positive mode. An internal standard (d4-NAM to NAM and N-methyl-NAM) was added to 25 µL of mouse plasma. Chromatographic separation was run (0.5 mL/min) using an InfinityLab Poroshell 120 HILIC-Z column (2.7 µm, 2.1 × 100 mm, Agilent) and a gradient mobile phase consisting of a solution of 50 mM ammonium acetate with 5 µM acid medrionic (InfinityLab deactivator additive, Agilent) as additive (Phase A) and acetonitrile (Phase B) were used. The mass spectrometer parameters were: drying and sheath gas temperatures 290ºC and 400ºC, respectively; source and sheath gas flows 20 and 12 L/min, respectively; nebulizer flow 60 psi; capillary voltage 2,500 V; nozzle voltage 500 V; and iFunnel HRF and LRF 110 and 80V, respectively. QqQ worked in MRM mode using the transitions that can be seen in **Table S1**.

*Analysis and quantification of aortic lesion*

Proximal aortic atherosclerotic lesions in mice were evaluated in serial cryosections of optimal cutting temperature (OCT) compound-embedded preparations at the end of the experiment. Mice hearts were flushed with saline, embedded in OCT, and snap-frozen in dry ice. Frozen sections of 5-8 µm thickness were taken in the region of the proximal aorta starting from the end of the aortic sinus, and for 300 μm distally, according to the technique of Paigen et al. [3]. Cryosections were stained with Oil Red O (ORO) for lipids and the lesion area (surface area stained with ORO) and counterstained with hematoxylin. The lesion area was quantified using AxioVision image analysis software (Zeiss, Jena, Germany). Quantitative analysis of lipid-stained lesions was performed on sections starting at the end of the aortic sinus. The lipid-stained lesions were measured by digitizing morphometry and reported as area (µm^2^ per lesion per mouse) using Image J software.

*Evaluation of the antioxidant effect of NAM on oxidizability of plasma ApoB-containing lipoproteins*

The susceptibility of mouse lipoproteins to copper-induced lipid oxidation and the capacity of NAM to inhibit the oxidative modification of human LDL were measured by monitoring the formation of conjugated dienes using a BioTek Synergy HT spectrophotometer (BioTek Synergy, Winooski, VT, USA). Both non-HDL and LDL were respectively isolated from plasma-EDTA by sequential ultracentrifugation at 100,000 *g,* using potassium bromide for density adjustment, for 24 h, with an analytical fixed-angle rotor (50.3, Beckman Coulter) or preparative fixed-angle rotor (70.38, Beckman Coulter, Fullerton, CA), as appropriate. Mouse non-HDL (density range <1.063 g/mL) were obtained from independent plasma pools composed of a minimum of 2-3 mice/experimental group, whereas human low-density lipoproteins (LDL, 1.019<d<1.063 g/mL) were from normolipidemic volunteers. The obtention and use of human plasma samples for this study were approved by the Ethics Committee of the Hospital de la Santa Creu i Sant Pau in Barcelona. Isolated non-HDL or LDL were dialyzed in phosphate-saline buffer at pH 7.4 (PBS) by gel filtration on PD-10 columns (Sephadex G-25™ M, cat#17-0851-01; GE Healthcare, Little Chalfont, UK) prior to oxidation, respectively. Oxidation was initiated by adding either 5 µM copper (II) sulfate (CuSO_4_) to PBS-dialyzed human LDL (0.1 g/L ApoB) or 2.5 µM CuSO_4_ to mouse non-HDL (0.1 mmol/L phospholipids), as appropriate. Conjugated diene kinetics were obtained by continuously monitoring the absorbance conjugated dienes at a λ of 234 nm at 37°C for 6 hours using a BioTek Synergy HT spectrophotometer (BioTek Synergy, Winooski, VT, USA). The lag phase was calculated from the intersection point between the maximal slope of the curve and initial absorbance, as previously described [4].

Serum oxidized LDL (oxLDL) levels were determined using a murine oxLDL sandwich ELISA (cat# SEA527Mu-96T, Cloud-Clone Corp, Houston, TX).

*NAM-mediated prevention of lipopolysaccharide (LPS)-induced inflammation in cultured macrophages*

Mouse macrophages J774A.1 stimulated with lipopolysaccharide (LPS) (from *E.coli* O111:B4; cat#L-4391-1MG, Merck KGaA, Darmstadt, Germany) were incubated with different concentrations of NAM to test the relative mRNA levels of different cytokines in cell extracts by real time qPCR. Briefly, J774A.1 macrophages (ATCC® TIB-67™) were cultured in 6-well flat-bottom cell culture plates at 1×10^6^ cells/well in growth medium containing RPMI 1640 medium supplemented with 2 mM glutamine, 100 U/L of penicillin/streptomycin, 5% fetal bovine serum at 37°C in an atmosphere of 5% CO_2_. Cultured macrophages were stimulated with 0.1 µg/mL of LPS for 24 h. After 24 h, the medium was removed, and fresh growth medium was added in the absence (untreated) or presence of different final concentrations of NAM, ranging from 1-10 mM for additional 24 h. In each assay, each experimental condition was assayed in duplicate. After 24 h of exposition cell culture, gene expression was analyzed. This experiment was repeated up to 4 times.

Effect of NAM on cholesterol efflux capacity by HDL

Cellular cholesterol efflux induced by HDL was determined in vitro using [^3^H]-cholesterol-labeled J774A.1 mouse macrophages (ATCC® TIB67™, Manassas, VA) as described [5]. Briefly, 200,000 cells/well were seeded in 6-well plates and allowed to grow for 24 h in RPMI 1640 medium containing 2 mM L-Glutamine (Sigma) complemented with 10% fetal bovine serum (FBS) (Merck KGaA, Darmstadt, Germany) and 100 U/mL penicillin/streptomycin (Merck KGaA, Darmstadt, Germany). After that, cells were labeled for 60 h in the presence of 1 µCi/well of [1α,2α(n)-3H]cholesterol (GE Healthcare, Little Chalfont, UK) and 5% FBS. The cells were then equilibrated with 0.2% bovine serum albumin (BSA) in medium overnight and incubated for 4 h with human HDL (density 1.063-1.210 g/mL) (50 µg/mL cholesterol), previously isolated by sequential ultracentrifugation and dialyzed in phosphate-buffered saline, as described above. Radioactivity was measured in both the medium and the cells, and the percentage of cholesterol efflux was calculated.

*Quantitative real-time RT-PCR analyses*

The RNA from cultured macrophages was isolated using TRIzol® reagent (cat#15596018, Ambion by Life Technologies, Madrid, Spain) following the manufacturer’s protocol and purified using the EZ-10 DNAaway RNA Kit de miniprep (cat#BS88136, BIO BASIC by Cliniscience, S.L., Madrid, Spain). RNA concentration was determined as above using a NanoDrop2000 (Thermo Scientific, Barcelona, Spain). Total RNA was reverse-transcribed using anchored Oligo (dT)_23_ (cat#O4387, Merck KGaA, Darmstadt, Germany) and a mixture of dNTPs (cat#D7295, Merck KGaA, Darmstadt, Germany), and M-ML reverse Transcriptase, RNase H Minus, and Point Mutant (cat#M3683, Promega Biotech Ibérica, Madrid, Spain) to generate cDNA. cDNA was subjected to fluorescence-based quantitative real-time PCR (qPCR) using Taqman Master Mix (cat#A6102, Promega Biotech Ibérica, Madrid, Spain). Thermal cycling conditions included 10 min at 95°C before the onset of the PCR cycles followed by which consisted of 40 cycles at 95°C for 15 s, and 65°C for 1 min. Specific mouse Taqman probes (Applied Biosystems by Life technologies Madrid, Spain) were used to analyze gene expression in mouse tissues (**Table S2**). Real-time qPCR assays were performed on a CFX96 Touch Real-Time PCR Detection System (Bio-Rad Laboratories SA, Life Science Group, Madrid, Spain). All analyses were performed in duplicate. As an internal control, gene expression was normalized to the mouse β-actin, i.e., *Actb* (Mm00607939_s1) (reference gene). The relative mRNA expression levels were calculated using the ΔΔCt method.

Aortic tissue was homogenized with Precellys® Evolution connected to a Cryolys cooling unit (Bertin Technologies, Aix-en-Provence, France). Total RNA was extracted using TRI reagent solution (cat#T9424, Merck KGaA, Darmstadt, Germany) following the manufacturer's recommendations. RNA concentration was determined as above using a NanoDrop2000 (Thermo Scientific). cDNA synthesis was performed from 1 µg total RNA of each sample by using anchored oligo(dT)15 (cat#C1101, Promega Biotech Ibérica, Madrid, Spain), using 200 units of M-MLV reverse transcriptase RNase H Minus, Point Mutant (#M3681, Promega), and 10 mM dNTPs (cat#U1511, Promega), following manufacturer’s recommendations. Fluorescence-based quantitative real-time PCR (qPCR) was performed using a CFX384^TM^ Real-Time Detection System (Bio-Rad Laboratories SA, Life Science Group). The temperature cycle was 95ºC for 10 min, followed by 35 cycles of 95ºC for 30 sec, 60ºC for 30 sec, and 72ºC for 30 sec. Analysis was performed in technical triplicates using the Power SYBR Green Reagent Kit (cat#4367659, Applied Biosystems, Life Technologies, Madrid, Spain) following manufacturer’s instructions. The Power SYBR® Green PCR Master Mix is supplied in a 2-fold concentration, which contains the SYBR® Green I Dye, AmpliTaq Gold® DNA Polymerase, UP, dNTPs, and optimized buffer components and optimized for SYBR® Green reagent reactions. The oligonucleotide sequences for the primer pairs used are described in **Tables S3 and S4**. The MIQE guidelines (https://academic.oup.com/clinchem/article/55/4/611/5631762) were followed when we designed the primers used in the current study. In most cases, we used Primer3web software to design the primers ourselves. In other cases, we used validated primers obtained from the PrimerBank database (ID are specified in **Table S4**). Primer blast was used for the in silico specificity screen, and specificity was further validated by agarose gel electrophoresis and by analyzing the melting curve for each set of primers. The primers were designed after considering low self-complementarities, as well as melting temperatures suitable for an annealing temperature of 60ºC during the qPCR analysis. In all cases, the Cq values of nontemplate controls for each primer set were below the limit of detection. As recommended by the MIQE guidelines, essential data, including the amplicon length, the exon to which each primer binds, and the transcript variants recognized by each primer set, are shown in **Table S4**. Data were expressed as relative mRNA levels normalized to ribosomal *L14* expression.

*Immunohistochemistry*

Thoracic aorta segments from untreated animals (n = 6), treated with NAM at low dose (n=7) and high dose (n=10) were fixed in Formalin solution, neutral buffered 10% (cat#HT501128, Merck KGaA, Darmstadt, Germany). Seven-micrometer sections of paraffin-embedded tissue samples were incubated with mouse monoclonal antibodies against IL-10 (cat# GTX632359, diluted 1:50, v:v) and rabbit polyclonal antibodies against TNFα (cat# GTX110520, diluted 1:200, v:v) from GeneTex and stained with diaminobenzidine (DAB) in a Dako Autostainer Link 48 using the Dako EnVision+System-HRP-DAB-kit, according to the manufacturer’s protocol. Briefly, primary antibodies were incubated at 4°C for 30 min in the presence of mouse (IL-10) or rabbit (TNFα) linkers, respectively. The labeled polymer was applied for 30 min, and staining was visualized after a 20-min exposure to diaminobenzidine (DAB) as chromogen. Slides were then dehydrated and coverslipped and images were obtained using a BX61 Olympus bright-field microscope. Images were quantified using ImageJ-Fiji software. A macro for deconvolution of hematoxylin and DAB staining and a binary mask was performed. Only the DAB mask was considered for free delimitation of vessel area fraction quantification. Three microphotographs under similar magnification (x20; scale bar 100 μm adjusted for pixel) were recorded for each preparation. Three different areas for each microphotography were quantified.

*Statistical methods*

The data are presented as medians (interquartile ranges). Statistical analyses were performed using GraphPad Prism software (GPAD, version 5.0, San Diego, CA, USA). The effects of NAM administration on gross and biochemical parameters, histological traits, and gene expression levels were determined using a nonparametric Kruskal-Wallis test followed by the Dunn multiple comparison test. Spearman’s rho correlation coefficients were calculated to determine the correlations between atherosclerosis and parameters of lipoprotein function, with all parameters considered as nonparametric variables. Differences between groups were considered statistically significant when the P value was <0.05.

[1] Mendez-Lara, K.A.; Santos, d.; Farre, N.; Ruiz-Nogales, S.; Leanez, S.; Sanchez-Quesada, J.L.; Zapico, E.; Lerma, E.; Escola-Gil, J.C.; Blanco-Vaca, F.; Martin-Campos, J.M.; Julve, J.; Pol, O. *PLoS One* **2018**, *13*, e0204841.

[2] Hara, A.; Radin, N.S. *Anal. Biochem*. **1978**, *90*, 420.

[3] Paigen, B.; Morrow, A.; Holmes, P.A.; Mitchell, D.; Williams, R.A. *Atherosclerosis* **1987**, *68*, 231.

[4] Ribas, V.; Sanchez-Quesada, J.L.; Anton, R.; Camacho, M.; Julve, J.; Escola-Gil, J. C.; Vila, L.; Ordonez-Llanos, J.; Blanco-Vaca, F. *Circ. Res.* **2004**, *95*, 789.

[5] J.C. Escola-Gil, J. C.; Lee-Rueckert, M.; Santos, D.; Cedo, L.; Blanco-Vaca, F.; Julve, J. *Methods Mol. Biol*. **2015**, *1339*, 211.
